# Effect of Efavirenz on Endogenous Progesterone Concentrations and Contraceptive Outcomes among Ugandan HIV Infected Women Coadministering Ethinylestradiol/Levonorgestrel

**DOI:** 10.1155/2017/6531709

**Published:** 2017-07-31

**Authors:** Grant Munkwase, Kuteesa R. Bisaso, Othman Kakaire, Sarah Nanzigu

**Affiliations:** ^1^Department of Pharmacology and Therapeutics, College of Health Sciences, Makerere University, P.O. Box 7062, Kampala, Uganda; ^2^Department of Obstetrics and Gynaecology, College of Health Sciences, Makerere University, P.O. Box 7062, Kampala, Uganda

## Abstract

This study assessed the effect of efavirenz mid-dose plasma concentrations on mid-luteal endogenous progesterone concentrations and contraceptive outcomes among 49 HIV infected women coadministering ethinylestradiol/levonorgestrel, including 34 HIV positive women on Highly Active Antiretroviral Therapy (HAART) and 15 HAART naïve HIV infected women, purposively selected from Mulago Hospital, Uganda. A blood sample was collected once between days 20 and 22 of each woman's menstrual cycle for measuring endogenous progesterone and efavirenz concentrations by electrochemiluminescence technology and High Performance Liquid Chromatography (HPLC), respectively. Descriptive statistical analysis and correlation and logistic regression analysis were done using SPSS v.21 and R3.1. Efavirenz showed a weak positive linear relationship with endogenous progesterone at efavirenz concentrations below 12 *μ*g/ml. Based on serum endogenous progesterone, the observed hormonal contraceptives failure rate (24.5%) was higher than expected (maximum 8%). A higher proportion of HIV positive women on efavirenz based HAART (26.5%) was at risk of contraceptive failure than their HIV infected HAART naïve counterparts (20%) though it was not statistically significant (*p* = 0.63). Efavirenz mid-dose plasma concentrations seem to have no significant effect on mid-luteal endogenous progesterone concentrations and contraceptive outcomes among HIV infected Ugandan women coadministering ethinylestradiol/levonorgestrel oral pills.

## 1. Introduction 

The disproportionate high prevalence of HIV/AIDS among very fertile women in sub-Saharan Africa (SSA) has been associated with a high burden of vertical transmission of HIV [1, 2]. According to a 2013 UNAIDS report, 24.7 million people were living with HIV/AIDS in SSA, accounting for 71% of the global burden, with 58% of all HIV/AIDS cases in SSA having been reported among women [3]. One strategy of lowering mother to child transmission of HIV is effective contraception [4]. Hormonal contraceptives (HCs) including oral contraceptive pills are known to be the most efficient and reliable contraceptive methods. Oral contraceptive pills are the second most used family planning method after injectable contraceptives in Uganda among the hormonal contraceptives [5, 6]. Ethinylestradiol/levonorgestrel containing pills are the most used pills in Uganda.

With the most recent guidelines of initiating everybody who tests HIV positive on Highly Active Antiretroviral Therapy (HAART), nearly all HIV reproductive women are on HAART [7, 8]. Most HAART regimens include a nonnucleoside reverse transcriptase inhibitor (NNRTI), mainly efavirenz or nevirapine. Efavirenz is a preferred NNRTI in many countries including Uganda because of its once daily dosing, cost effectiveness, and better safety profile [9].

Both efavirenz and ethinylestradiol/levonorgestrel undergo extensive hepatic metabolism. Efavirenz undergoes hepatic biotransformation mainly by CYP2B6 and CYP3A4 [10, 11]. Ethinylestradiol/levonorgestrel on the other hand is metabolized mainly by CYP3A4 and UGT1A4 [12–14]. Women coadministering efavirenz and ethinylestradiol/levonorgestrel are at a risk of potential drug interactions. The interactions may be pharmacokinetic (PK) or pharmacodynamic (PD) in nature. A number of pharmacokinetic studies have examined interactions between antiretroviral medications and hormonal contraceptives, but less is known about the PD interactions. Previous PK studies done among healthy volunteers have shown changes (either increases or decreases) in levels of ethinylestradiol and the progestin in combination pills, among women who are taking certain protease inhibitors (PIs) or nonnucleoside reverse transcriptase inhibitors (NNRTIs) including efavirenz [15–18]. Two of the three PD studies done in Netherlands and Malawi showed efavirenz to be associated with unfavorable endogenous progesterone levels when coadministered with combined pills [16, 19], while one study reported no effect of efavirenz on endogenous progesterone levels [15]. There are currently no reported interactions between hormonal contraceptives and nucleoside analogues, integrase inhibitors, or CCR5 antagonists [20]. All the previous studies were done among HIV negative volunteers and among HIV positive noncontraceptive users who were then initiated on efavirenz or oral pills. However, this study explored the situation in real life conditions among Ugandan women.

## 2. Materials and Methods

### 2.1. Study Design and Participants

An observational effectiveness study was conducted at The AIDS Support Organization (TASO), an HIV care centre at Mulago National Referral Hospital in Uganda between October 2015 and March 2016. Mulago Hospital is Uganda's national referral hospital located in Kampala district. Ethical approval was obtained from Makerere University School of Biomedical Sciences Higher Degrees Research and Ethics Committee (SBS-249), Uganda National Council for Science and Technology (HS1815), TASO Research and Ethics Committee (TASO/ADC04/15-UG-REC-009). The study was done in accordance with the principles of declaration of Helsinki and informed consent was obtained from the study participants.

Forty-nine women aged 15–49 years were purposively selected from women receiving outpatient care at TASO, the HIV care centre at Mulago Hospital. These included 34 HIV positive women on efavirenz based HAART and 15 HAART naïve HIV positive women using combined oral pills who provided informed consent. Purposive sampling was used because of the scarcity of participants. The study excluded pregnant women, women with irregular menstrual cycles, women on HAART regimens excluding efavirenz, women who had used injectable contraceptives or implants within the previous 6 months, and women coadministering rifampicin, griseofulvin, carbamazepine, phenobarbitone, phenytoin, and modafinil at the time of enrollment. All the study participants were screened for pregnancy at enrollment.

### 2.2. Variables

The predictor variable was serum efavirenz concentrations, while the outcome variable was serum endogenous progesterone concentrations.

### 2.3. Blood Sample Collection and Laboratory Analysis

About 4 mls of blood was drawn once during mid-luteal phase (20th day–22nd day) of each woman's menstrual cycle, 12–15 hours since the last dose of efavirenz (if on HAART) or the oral contraceptive pill (if not on HAART), from participants who met the inclusion criteria and had provided informed consent. The blood sample was taken as whole blood to the Uganda Cancer Institute (UCI) Laboratory at Mulago for processing of plasma. About one ml of plasma was used for measurement of endogenous progesterone at UCI Laboratory, while the remaining plasma was used for measurement of serum efavirenz concentration at Pharmacokinetics Laboratory, Department of Pharmacology and Therapeutics, Makerere University.

Plasma was prepared from blood by centrifugation at 3000*g* for 10 minutes. A portion of plasma (1.0 ml) was used for progesterone measurement on the same day of sample collection and the remaining portion (about 2.0 mls) was stored at −60°C until HPLC analysis was performed.

The endogenous progesterone estimation was done by electrochemiluminescence technology using a fully automated, random access system Cobas e 411 analyzer. Efavirenz concentrations were determined using reversed phase High Performance Liquid Chromatography (HPLC) with UV detection [21]. The HPLC 10A instrument from Shimadzu manufacturing Inc., Japan, was used in the analysis. The column used was Ace3C18, 3 *μ*m 50 × 3.0 mm. The mobile phase consisted of 30% acetonitrile, 30% methanol, 4 mM potassium hydroxide, and 10 mM acetic acid with pH adjusted to 4.3. Plasma proteins were precipitated with acetonitrile before centrifuging. The supernatant (100 *μ*l) was eluted at 0.8 ml/min for five minutes. The retention time for efavirenz was 3.5 minutes as detected at UV-VIS 1,210 nm, UV-VIS 2,220 nm. The method was linear with the limit of quantification set at 0.4 *μ*g/ml. The interday coefficient of variation was 5.4%.

### 2.4. Statistical Analysis

Data analysis was done using SPSS v.21 and R.3.1. Mean and standard deviation were computed for age, weight, progesterone concentrations, and efavirenz concentrations. An independent samples* t*-test was also done to assess whether HIV positive women on HAART were significantly different from HAART naïve HIV positive women. Serum endogenous progesterone concentration served as a surrogate marker for ovulation and a cutoff point of 10.0 ng/ml of endogenous mid-luteal progesterone concentration was used in predicting whether ovulation had occurred or not for logistic regression analysis. At concentrations above 10.0 ng/ml, we were certain that ovulation took place [15, 22, 23].

Exploratory data analysis was done using R. 3.1.0. A scatter plot of progesterone concentrations against efavirenz concentrations was generated and analyzed for the trend of relationship using locally weighted smoothing (LOWESS). The generalized additive modeling method (using the gam package) was used to estimate the nature of the relationship and predict expected values of progesterone. Diagnostic plots (dependent variable versus individual predictions) and (individual residuals versus individual predictions) were utilized to diagnose goodness of fit of the model to the data. The final model was used to estimate the function and significance of relationship between progesterone and efavirenz.

## 3. Results 

This study enrolled 49 women (including 34 HIV positive women on HAART and 15 HAART naïve HIV positive women). There was no statistically significant difference between HIV positive women on HAART and HAART naïve HIV positive with regard to their age, weight, and progesterone concentrations. HIV positive women on HAART regimen had a nonsignificant higher mean endogenous progesterone concentration (6.93 ng/ml) than HAART naïve HIV positive women (5.79 ng/ml) (*p* = 0.57) (Table 1). The mean efavirenz concentration in HIV positive women on HAART was 14.56 *μ*g/ml with a standard deviation of 13.70 *μ*g/ml and a range of 1.38 *μ*g/ml–58.79 *μ*g/ml.

### 3.1. Contraceptive Outcomes among HIV Infected Women Taking Combined Oral Contraceptive Pills between October 2015 and March 2016 at Mulago Hospital

Mid-luteal endogenous progesterone concentration was used as a surrogate marker for ovulation and was taken as the measure of contraceptive outcome.

At a cutoff of 10.0 ng/ml of endogenous progesterone, the proportion of HIV positive women on HAART at risk of contraceptive failure was 26.5%, while that for HAART naïve HIV positive women was 20% and it was not statistically significant (OR = 1.4, 95% CI = 0.33–6.31, *p* = 0.63) (Table 2).

### 3.2. Assessment of Relationship between Endogenous Progesterone Concentration and Other Predictor Variables in the Study Including Efavirenz Concentration

**Figure 1:**
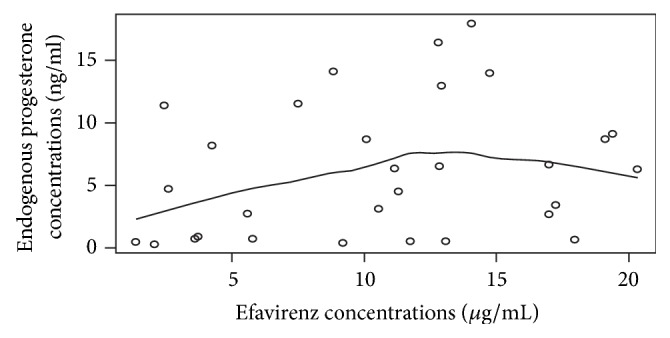
Variation of endogenous progesterone concentrations with efavirenz concentrations for 30 HIV infected women coadministering efavirenz and oral pills. Four women on HAART (outliers) were excluded in this analysis. There is a nonsignificant positive linear relationship between progesterone and efavirenz for efavirenz concentrations below 12 *µ*g/ml. Above 12 *µ*g/ml of efavirenz, progesterone begins to fall though it is not significant.

From exploratory data analysis, the trend showed that, for efavirenz concentrations less than 12 *μ*g/ml, there was a positive linear relationship between progesterone concentrations and efavirenz concentrations. The relationship seemed to disappear at efavirenz concentrations above 12 *μ*g/ml, though it was not significant (Figure 1).

**Figure 2:**
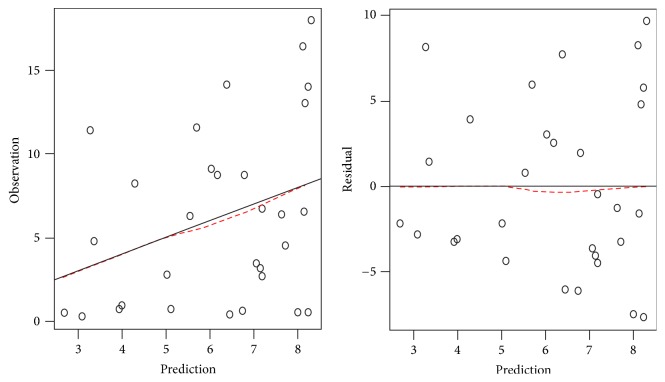
Goodness of fit plots. Both plots indicate that the model adequately fitted the data.

The selected generalized additive model adequately fitted the data as shown by the diagnostic plots (Figure 2). There was no significant relationship between the nonparametric effects of efavirenz and progesterone (*F* = 1.096, *p* = 0.34).

### 3.3. Factors Influencing Endogenous Progesterone Levels and Contraceptive Outcomes

Age, weight, interval between drug administration and sampling, number of days since LNMP, and efavirenz concentrations were evaluated for their effect on contraceptive outcomes (failure or success) based on endogenous progesterone, in binary logistic regression analysis. Variables were included in the model using a stepwise “enter” method, and the full model was assessed for goodness of fit using the Hosmer and Lemeshow test. Hosmer and Lemeshow test showed a Chi-square value of 11.48 and *p* value of 0.18 which indicated that the model was a good fit for the data. Sampling on day 21 was the only variable that significantly influenced progesterone concentrations and hence contraceptive outcomes. Women who were sampled on day 21 were twenty-one times more likely to be at risk of contraceptive failure than women sampled on day 22 (OR = 21.3, *p* = 0.04) (Table 3).

## 4. Discussion 

This study aimed at assessing the effect of efavirenz based HAART regimen on endogenous progesterone and contraceptive outcomes among HIV positive reproductive women who were coadministering ethinylestradiol/levonorgestrel oral pills at Mulago Hospital.

The relationship between efavirenz and endogenous progesterone was only linear for efavirenz concentrations less than 12 *μ*g/ml. The progesterone concentrations observed among HIV positive women on efavirenz based HAART regimens seemed to be independent of efavirenz concentrations. In fact, the mean endogenous progesterone concentrations were similar between HIV positive women on HAART and HAART naïve HIV positive women (6.9 ng/ml and 5.8 ng/ml, resp., *p* = 0.57). This finding is in agreement with a study done among HIV negative volunteers who coadministered efavirenz and ethinylestradiol/norgestimate and found that efavirenz had no effect on endogenous progesterone [15].

On the other hand, a study that reported an effect of efavirenz on endogenous progesterone was based on comparison with progesterone levels observed in women administering nevirapine, the other NNRTI [16]. The best comparison should be made between women using oral pills alone and women using both pills and efavirenz.

Sampling on day 21 was associated with a higher risk of contraceptive failure among women using oral pills when compared to sampling on day 20 or day 22. Day 21 has been reported in the literature as the day for peak endogenous progesterone concentrations in women with regular menstrual cycles [24]. Therefore, women with regular menstrual cycles who were sampled on day 21 were likely to report peak progesterone concentrations. The higher the progesterone concentration, the greater the possibility of ovulation and the higher the risk of contraceptive failure.

HIV positive women using efavirenz had a nonsignificant slightly higher risk of contraceptive failure than HAART naïve HIV positive women (26.5% versus 20%). The slightly higher risk of failure among HIV positive women administering efavirenz may be attributed to the inductive effect of the nucleoside analogue on CPY450 system, particularly CYP3A4 [25, 26]. The observed risk of contraceptive failure on oral pills based on endogenous progesterone in both groups of women was about three times higher than the 8% associated with typical use. Use of endogenous progesterone alone, as a biomarker for ovulation and as the measure for contraceptive outcome, may have contributed to overestimation of the risk of contraceptive failure. This may stem from lack of a clear cutoff point for which one may be certain that ovulation took place. Different countries and laboratories have different cutoff points for the endogenous progesterone concentrations indicative of ovulation. Furthermore, progesterone is secreted in a pulsatile nature and it fluctuates so many times a day. In addition, there are additional factors after ovulation, including “egg quality,” which may affect outcomes of pregnancy, and all these are often not taken into account.

Failure on oral pills in this study may have been contributed to by poor adherence since women admitted having been taking oral pills poorly at the time of unwanted pregnancy. They would take the routine pills only before or after having sex. Such women need to be educated on the best way they can utilize oral pills and also make them aware of the emergency pills. Poor adherence to oral pills among the HIV infected women may partly be due to the pill burden since they are on daily cotrimoxazole prophylaxis and/or HAART regimens.

This study had some limitations. Serum level of endogenous progesterone alone was the outcome measure of contraceptive effectiveness and therefore did not take into account other mechanisms that may contribute to contraceptive effectiveness. We did not measure the serum concentrations of ethinylestradiol/Levonorgestrel and thus we could not explore the relationship between the pharmacokinetics and pharmacodynamics of the concerned contraceptive in the face of efavirenz coadministration. The single-time sampling of blood to measure endogenous progesterone limited our precision of progesterone measurements due to the pulsatile nature of secretion of progesterone

## 5. Conclusions 

Efavirenz seems to have no significant effect on endogenous progesterone and contraceptive outcomes among the studied HIV positive Ugandan women coadministering ethinylestradiol/levonorgestrel oral pills. There was also a three times higher risk of contraceptive failure for oral pills among Ugandan HIV positive women when compared to their typical use failure rate. Further exploration of this concern is warranted in a larger population of women given the high fertility rate in the country.

## Figures and Tables

**Table 1:** Comparison of variables between HIV positive women on HAART and HAART naive HIV positive women taking combined oral contraceptive pills between October 2015 and March 2016 at TASO Clinic, Mulago Hospital.

Variable	HIV positive women on HAART (*n* = 34)	HAART naive HIV positive women (*n* = 15)	*t*	*p* value
Mean age in yrs (SD)	29.7 (6.51)	27.3 (3.95)	1.29	0.20
Mean weight in Kgs (SD)	63.4 (10.99)	61.2 (6.53)	0.73	0.47
Mean interval^*ί*^ in hrs (SD)	13.2 (0.83)	13.1 (0.74)	0.39	0.70
Mean progesterone levels in ng/ml (SD)	6.9 (6.51)	5.8 (6.17)	0.57	0.57

^*ί*^Time between last dose of efavirenz or oral contraceptive and sampling; SD = standard deviation; Kgs = kilograms.

**Table 2:** Contraceptive outcomes among HIV positive women taking combined oral contraceptive pills at TASO Clinic, Mulago Hospital.

	Contraceptive outcome		OR (95% CI)	*p* value
	Failure% (*n*)	Success% (*n*)		
HAART naive HIV positive women	20.0 (03)	80.0 (12)	1.0	
HIV positive women on efavirenz based HAART	26.5 (09)	73.5 (25)	1.4 (0.33–6.31)	0.63

**Table 3:** Factors associated with progesterone levels among HIV positive women on efavirenz based HAART in binary logistic regression analysis.

Variable	OR	95% CI	*p* value
Efavirenz conc. (*µ*g/ml)	1.08	0.90–1.30	0.39
Age (yrs)	1.04	0.85–1.27	0.70
Weight (Kgs)	1.12	0.97–1.29	0.14
interval^*ί*^ (hrs)	0.71	0.22–2.32	0.57
Sday			
Sday (1)	7.50	0.34–167.78	0.20
Sday (2)	21.30	1.06–426.64	**0.04**

^*ί*^Time between last dose of efavirenz or COC and sampling; Sday = reference sampling day (day 22), Sday (1) = sampling day 20, and Sday (2) = sampling day 21 of the menstrual cycle.
